# A case of undifferentiated carcinoma of the pancreas mimicking main-duct intraductal papillary mucinous neoplasm (IPMN)

**DOI:** 10.1007/s00261-014-0326-3

**Published:** 2014-12-21

**Authors:** Yuichi Kawai, Rei Nakamichi, Noriko Kamata, Hideo Miyake, Masahiko Fujino, Shigeki Itoh

**Affiliations:** 1 Department of Diagnostic Radiology, Japanese Red Cross Nagoya Daiichi Hospital, 3-35 Michishita-cho, Nakamura-ku, Nagoya, Aichi 453-8511 Japan; 2 General and Gastroenterological Surgery, Japanese Red Cross Nagoya Daiichi Hospital, Nagoya, Japan; 3 Department of Pathology, Japanese Red Cross Nagoya Daiichi Hospital, Nagoya, Japan

**Keywords:** Undifferentiated carcinoma of the pancreas, Main-duct intraductal papillary mucinous neoplasm, Intraductal tumor growth, Main pancreatic duct dilatation

## Abstract

We report here a rare case of undifferentiated carcinoma of the pancreas mimicking main-duct intraductal papillary mucinous neoplasm. In an 80-year-old woman, an approximately 8-mm papillary mass was incidentally detected at the downstream edge of a dilatated main pancreatic duct lumen on CT and MRI. Main pancreatic duct dilatation in the pancreatic body and tail and parenchymal atrophy were observed in the upstream of the mass. Histopathologically, the tumor protruded into the downstream edge of the dilatated main pancreatic duct lumen in the pancreatic body. The tumor cells had highly atypical nuclei and abundant polymorphic structures, and showed positive staining for granulocyte colony-stimulating factor, which led to the diagnosis of undifferentiated carcinoma. A total of 13 cases of undifferentiated carcinoma with intraductal tumor growth have been reported to date. The case report by Bergmann et al. has been the smallest in histopathological specimen, and the present case is the smallest in size detected by radiological images. Since early undifferentiated carcinoma of the pancreas can resemble those of main-duct intraductal papillary mucinous neoplasm in cross-sectional images, we have to consider undifferentiated carcinoma in the differential diagnosis of the solitary and papillary mass with low contrast enhancement in early phase in the main pancreatic duct.

Undifferentiated carcinoma of the pancreas is a subtype of pancreatic ductal carcinoma [1]. In general, the tumor size is large at onset and many cases have wide-spread hematogenous metastasis and lymphogenous metastasis, which result in an unfavorable prognosis [2]. We experienced a rare case of undifferentiated carcinoma of the pancreas mimicking main-duct intraductal papillary mucinous neoplasm (IPMN), in that the tumor was small and showed intraductal growth into the main pancreatic duct on imaging. Histopathological finding of this case was interesting in the histogenesis of undifferentiated carcinoma. Herein, we report our case with discussion about differential diagnosis of the papillary mass in the main pancreatic duct.

## Case report

An 80-year-old woman underwent a medical examination for hematuria, and a left renal pelvic tumor was detected on computed tomography (CT). Meanwhile, a mass was incidentally found in the pancreatic body, and a close examination was conducted. Her past medical history was colon cancer (endoscopically resected), uterine leiomyoma, appendicitis, and hypertension, but her family history was unremarkable. In the laboratory examination, the white blood cell (WBC) count and tumor markers (carcinoembryonic antigen, carbohydrate antigen 19-9, and pancreatic cancer-associated antigen) were within the normal limits.

**Fig. 1:**
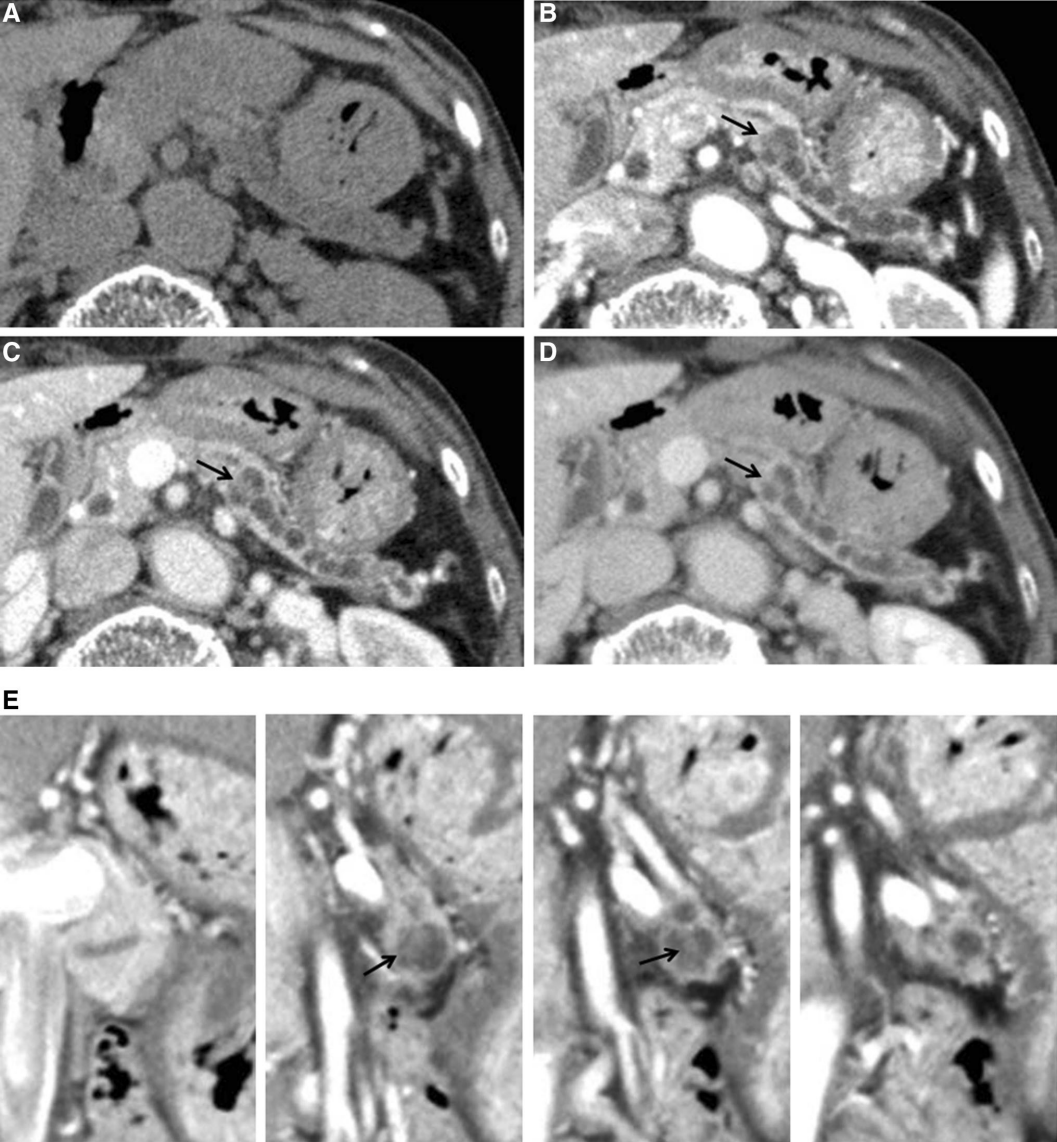
**A**–**E** Axial pre-enhanced (**A**) and axial multiphase dynamic contrast-enhanced (**B** pancreatic phase; **C** portal venous phase; **D** equilibrium phase) and coronal contrast-enhanced (**E** portal venous phase) CT images show a papillary mass at the downstream edge of the dilatated main pancreatic duct lumen (*arrows*). The mass is slightly enhanced between the pancreatic phase and delayed phase on a dynamic study. The duct is dilatated in the pancreatic body and tail, but not in the downstream of the mass. The surrounding pancreatic parenchyma in the body and tail is atrophic and its border with the mass is irregular.

**Fig. 2:**
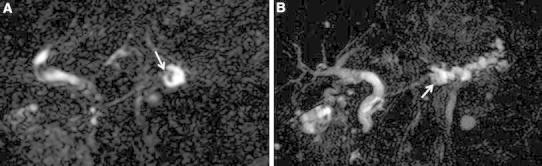
**A**, **B** MRCP (**A** original coronal 3D-MRCP; **B** 3D-MRCP MIP) images show a papillary mass with low signal intensity in the pancreatic body (*arrows*). Main pancreatic duct dilatation is observed in the body and tail, but not in the downstream of the mass.

**Fig. 3:**
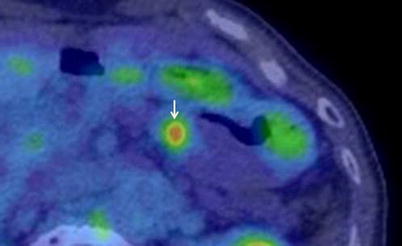
18-F FDG delayed PET/CT image shows uptake of FDG in accord with the papillary mass in the pancreatic body (*arrow*).

On CT, the main pancreatic duct was dilatated to 12 mm in largest diameter in the pancreatic body and tail, and an approximately 8-mm papillary mass was observed at the downstream edge of the dilatated duct lumen. The mass showed slight enhancement between the pancreatic phase and delayed phase on dynamic CT and its border with the surrounding parenchyma was irregular. The pancreatic parenchyma in the body and tail showed atrophy, with prolonged contrast enhancement. Main pancreatic duct dilatation was not seen in the downstream of the mass (Fig. 1). On magnetic resonance imaging, magnetic resonance cholangiopancreatography (MRCP) showed main pancreatic duct dilatation in the body and tail with a low signal intensity papillary mass in the downstream edge of the dilatated duct lumen. Main pancreatic duct dilatation in the downstream of the mass was not observed (Fig. 2). The 18F-fluorodeoxyglucose-positron emission tomography combined with computed tomography (18F-FDG-PET/CT) showed uptake of FDG in accord with the papillary mass in the pancreatic body. That increased in the delayed phase (maximum standardized uptake value (SUVmax): 4.4) compared with the early phase (SUVmax: 3.2) (Fig. 3).

Main-duct IPMN with an associated invasive carcinoma was suspected on the grounds that the dilated main pancreatic duct accompanied a solidary nodule, and distal pancreatectomy was performed. Meanwhile, left nephrectomy was performed for the left renal pelvic tumor.

**Fig. 4:**
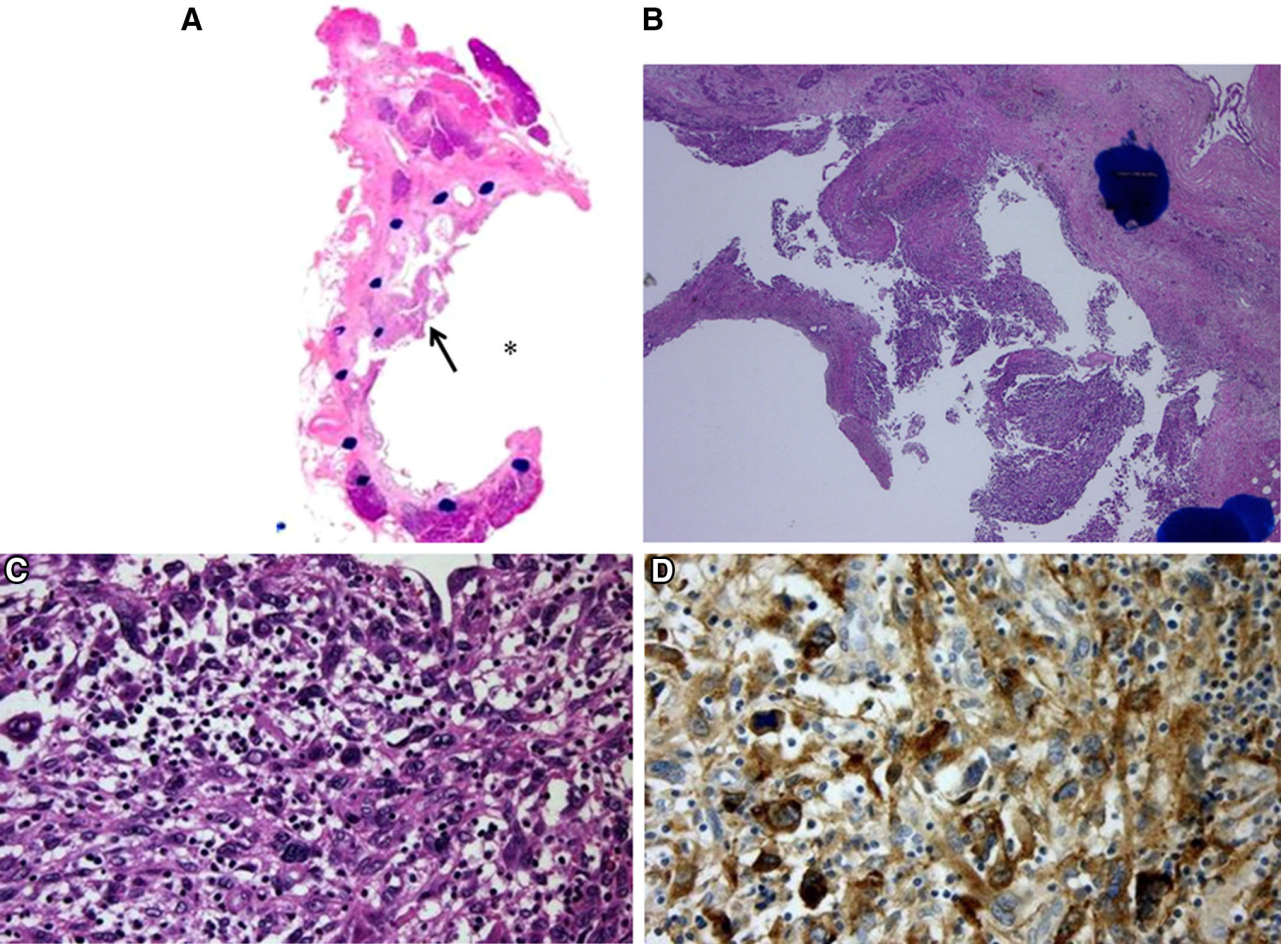
**A** Loupe findings show that the tumor (*arrow*) protrudes into the downstream edge of the dilatated main pancreatic duct lumen in the pancreatic body (*asterisk*). The maximum diameter of the papillate region of the tumor is 8 mm and the region of interstitial infiltrate is 7 mm. **B** H&E staining with low magnification shows the tumor invasion to the surrounding parenchyma and conspicuous interstitial growth with inflammatory cell infiltration. **C** H&E staining with high magnification shows highly atypical tumor cells with abundant scattered polymorphic structures. **D** The tumor cells show positive staining for G-CSF.

In the loupe findings, the tumor protruded papillary into the downstream edge of the dilatated main pancreatic duct lumen in the pancreatic body. The maximum diameter of the papillate region of the tumor was 8 mm and the region of interstitial infiltrate was 7 mm. The entire epithelium of the main pancreatic duct was substituted by atypical cells. Histopathologically, the tumor cells had highly atypical nuclei and abundant scattered polymorphic structures, and numerous multinucleated giant cells were also observed. The tumor cells showed positive staining for granulocyte colony-stimulating factor (G-CSF) (Fig. 4). The pathological diagnosis was undifferentiated carcinoma (pleomorphic type). The tumor slightly invaded the surrounding parenchyma associated with conspicuous interstitial growth and inflammatory cell infiltration. The pathological findings of IPMN were not detected. The left renal pelvic tumor was invasive urothelial carcinoma.

## Discussion

Undifferentiated carcinoma is a subtype of pancreatic ductal carcinoma that exhibits sarcomatous proliferation of severe mononuclear tumor cells and multinuclear giant cells. It accounts for 2–7% of all pancreatic cancers and frequently occurs in older men [2]. The favorite sites for this tumor are the pancreatic body and tail. It is classified into giant cell, pleomorphic cell, and spindle cell types in accordance with the cellular morphology, but they are often mixed [2, 3]. Abdominal pain, back pain, weight loss, fever, anorexia, and jaundice can be the initial symptoms [2]. About half of the patients show elevation of WBC count and C-reactive protein (CRP) for G-CSF production, and about 60% of the patients show elevation of CA19-9 [4]. Since the tumor is generally large at onset, and many cases have wide-spread hematogenous metastasis and lymphogenous metastasis, the prognosis is extremely unfavorable. On imaging, undifferentiated carcinoma typically shows heterogeneous and dense contrast enhancement that are superior in the peripheral zone and poor contrast enhancement in the center owing to hemorrhage or necrosis [5]

To the best of our knowledge, 13 cases of undifferentiated carcinoma with intraductal tumor growth into the main pancreatic duct have been reported to date [6–18]. Most cases were greater in size, and mainly showed mass formation in the pancreatic parenchyma with intraductal tumor growth into part of the main pancreatic duct [6–16]. However, Tezuka et al. reported the first case of undifferentiated carcinoma of the pancreas in situ within the main pancreatic duct [17] and a case report by Bergmann et al. showed 8 mm in the largest diameter pathologically [18]. When it comes to the case with detection in cross-section images, our case is the smallest in size in undifferentiated carcinoma with intraductal tumor growth into the main pancreatic duct.

Although the debate on the histogenesis of the tumor has been controversial, Bergmann et al. provided the evidence for a ductal origin of the tumor histopathologically [18]. Furthermore, Tezuka et al. reported the first case of undifferentiated carcinoma of the pancreas in situ within the main pancreatic duct, without evidence of invasion beyond the pancreatic duct [17]. In our case, the entire epithelium of the main pancreatic duct was substituted by atypical cells, but the papillate region of the tumor into the main pancreatic duct and the interstitial infiltrate of the tumor were small. This finding would support the origin of epithelial cells, not mesenchymal cells or stem cells. Additionally, some pathological reports have indicated that undifferentiated carcinoma in the early stage shows intraductal growth into the main pancreatic duct [6], and our radiological images capture the pathological features of the papillate region of the tumor in the early stage, however, the region of interstitial infiltrate cannot be detected.

IPMN and ITPN (intraductal tubulopapillary neoplasm) are categorized as intraductal pancreatic tumors, and are representative of pancreatic tumors with intraductal tumor growth into the main pancreatic duct [1]. Some cases of them occur multiply in the main pancreatic duct and the dilatation of the duct is prominent and diffuse [19, 20]. On the other hand, undifferentiated carcinoma mostly occurs solitary in the previous report and our case [6–18]. Acinar cell carcinoma and neuroendocrine tumor with intraductal tumor growth are also reported as rare cases [21–23]. They typically show high contrast enhancement in early phase on dynamic study, which can differentiate from undifferentiated carcinoma. Additionally, elevation of WBC count and CRP level can help the diagnosis of undifferentiated carcinoma [4]. However, in the cases with lack of these characteristics, differential diagnosis from early undifferentiated carcinoma would be difficult.

Since early undifferentiated carcinoma of the pancreas can resemble those of main-duct IPMN in cross-sectional images, we have to consider undifferentiated carcinoma in the differential diagnosis of the solitary and papillary mass with low contrast enhancement in early phase in the main pancreatic duct.

## Conclusions

We report a rare case of undifferentiated carcinoma of the pancreas mimicking main-duct IPMN. We have to consider undifferentiated carcinoma in the differential diagnosis of the solitary and papillary mass with low contrast enhancement in early phase in the main pancreatic duct.
